# The Pushing Sign for Early Skin Tethering in Breast Cancer

**DOI:** 10.7759/cureus.20471

**Published:** 2021-12-16

**Authors:** Vijay Naraynsingh, Johnathan K Jarvis, David M Milne, Shamir O Cawich, Dave Harnanan, Yardesh Singh, Shariful Islam

**Affiliations:** 1 Clinical Surgical Sciences, University of the West Indies, St. Augustine, TTO; 2 Surgery, Medical Associates Hospital, St. Joseph, TTO; 3 General Surgery, University of the West Indies, St. Augustine, TTO; 4 General Surgery, General Hospital Port of Spain, Port of Spain, TTO; 5 Surgery, University of the West Indies, St. Augustine, TTO; 6 General Surgery/Oncoplastic Breast Surgery, San Fernando General Hospital, San Fernando, TTO

**Keywords:** skin dimpling or puckering, skin tethering, pushing sign, early signs of breast cancer, breast cancer

## Abstract

Skin tethering (ST) is regarded as a classical clinical feature of breast cancer. In many cases, ST is not evident on inspection, with the arm raised and skin pinching over the lump. We have observed that pushing the lump in one or another direction may elicit skin dimpling that was not otherwise evident. In these cases, there is normal fat, grossly and histologically, between the tumor and the skin. Thus, the dimpling is not due to cutaneous infiltration. We believe that it is caused by tumor involvement of the ligaments of Cooper and present suggestions as to why it might be so. It may be that this is very early involvement of these ligaments, long before ST becomes very obvious. We report our experience with six such cases.

## Introduction

Skin tethering (ST) is a well-known clinical manifestation of breast cancer [1,2]. Some authors regard it as almost a pathognomonic disease. A review of the clinical findings of 802 symptomatic patients presenting to a breast clinic revealed that all cases who presented with skin tethering were subsequently diagnosed with breast cancer [3].

Skin "dimpling" or "puckering" is distinct from "tethering," as the former is the latter's presentation on inspection, which is a concavity or sunken appearance of the skin. Tethering is elicited on palpation of the lump in relation to the surrounding skin.

Fixation is the inability to "pinch" the skin over the lump, suggesting skin invasion. Browse defines fixation as a direct spread into the skin, which cannot be separated from the lesion in question [4].

The American Joint Committee on Cancer’s Staging Manual specifically states, "dimpling of the skin, tethering, and nipple retraction are caused by tension on Cooper's ligament(s), not by actual skin involvement" [5].

Adherence, attachment fixation, induration, and thickening are clinical evidence of extension to the skin or subcutaneous tissue [5]. ST is revealed on clinical examination when: (i) skin dimpling or puckering is present without any manipulation of the breast or chest wall; (ii) when the patient raises their hands over their head; and (iii) gently moving the lump in two planes, examining for wrinkling of the skin [6].

## Case presentation

Case 1

**Figure 1:**
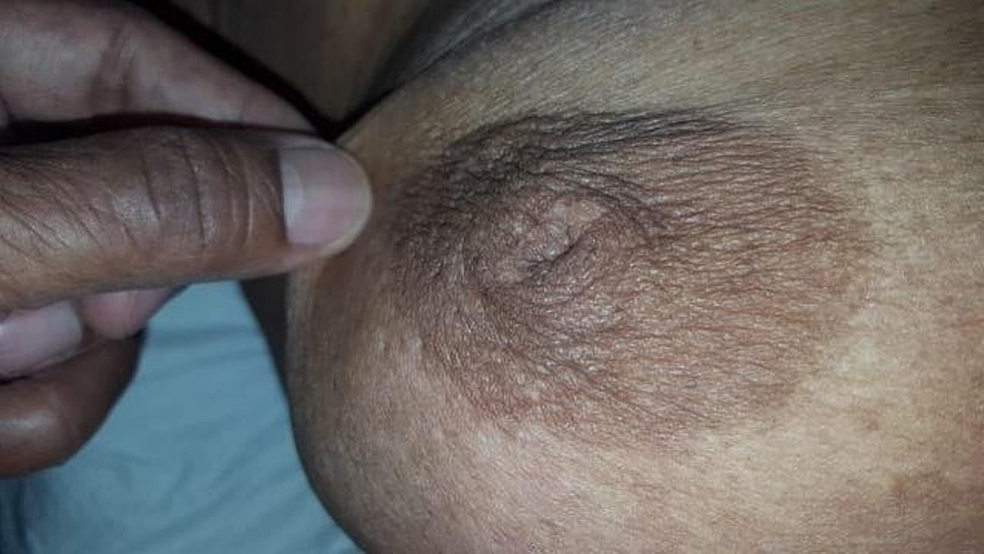
Skin pinched over the mass, demonstrating the absence of fixation

**Figure 2:**
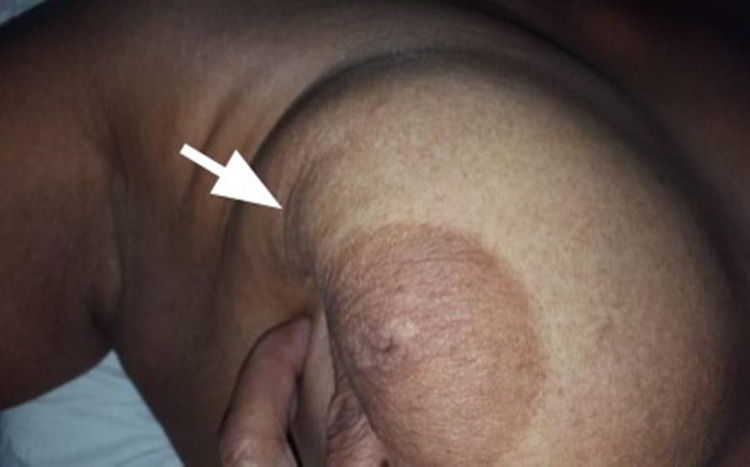
Pushing lump medially and upward demonstrates a concave depression on the overlying skin

**Figure 3:**
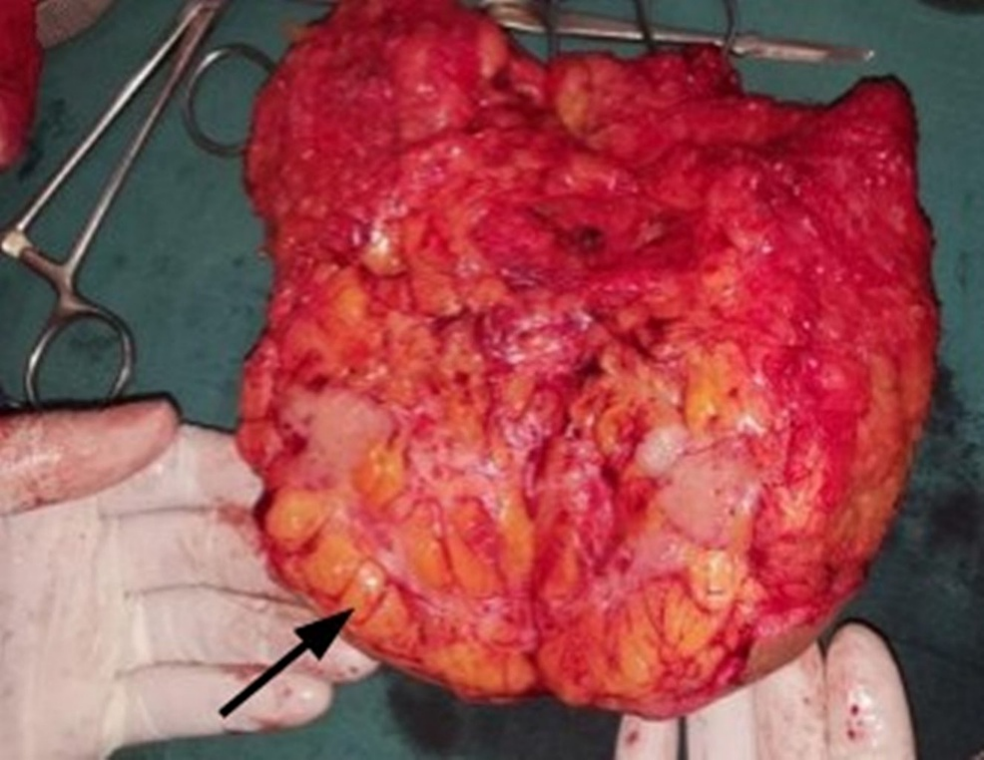
The cut surface of the tumor showed normal fat (arrow) between tumor and skin

A 62-year-old female presented with a 3 cm breast lump. The overlying skin was soft and could be easily pinched over the mass (Figure 1). However, on pushing the lump medially and upward, an obvious depression appeared on the overlying skin (Figure 2). After a mastectomy, the cut surface of the tumor showed normal fat between the tumor and skin, with no tumor invasion of the fat histologically (Figure 3).

Case 2

**Figure 4:**
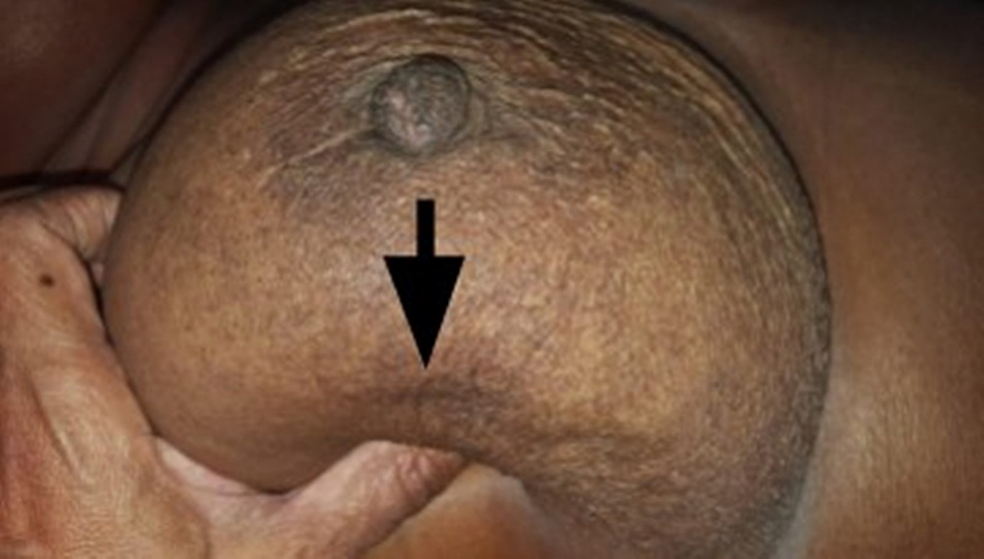
Pushing on lump shows depression on the overlying skin

A 68-year-old female presented with a 2.5 cm breast lump. It showed no features of skin tethering on inspection or arm elevation, and the overlying skin was very mobile and could be pinched over the lump. However, on pushing it, an obvious depression appeared in the overlying skin (Figure 4). Wide local excision with an ellipse of overlying skin showed invasive grade 2 carcinoma with ductal and lobular patterns. Neither the skin nor the subcutaneous fat had tumor invasion.

Case 3

**Figure 5:**
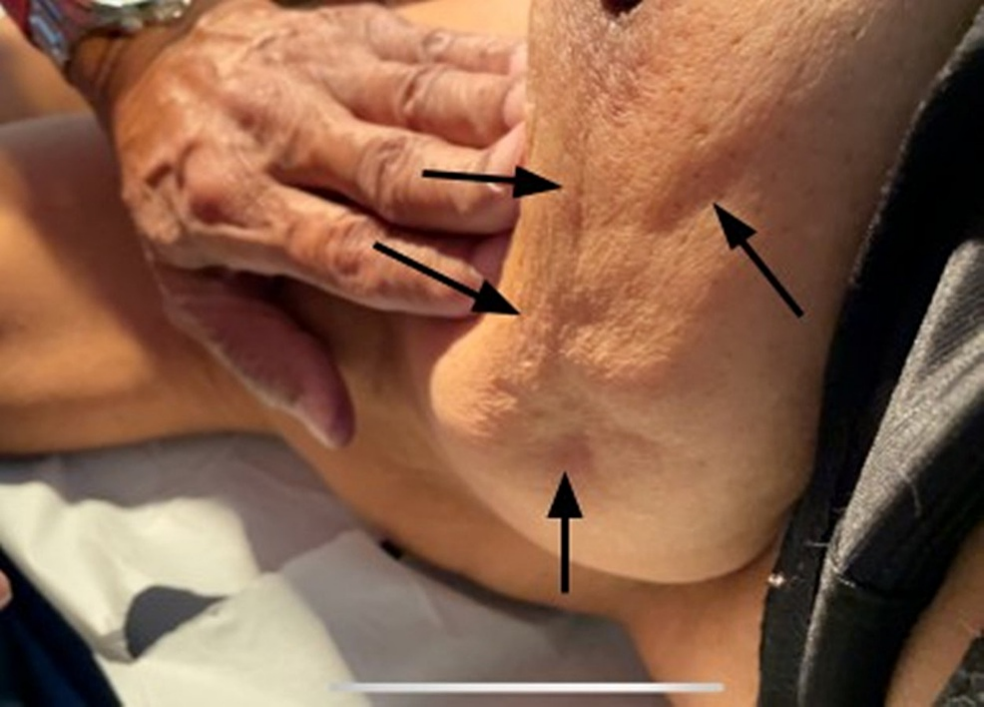
Pushing downward on the lump shows multiple depressions on the overlying skin

A 52-year-old female presented with a painless breast lump in the upper outer quadrant of her right breast. On examination, there was a 3 cm firm, mobile mass in the right breast. There was no peau d’orange, cutaneous edema, or skin tethering on inspection, arm elevation, or pinching of skin over the mass. Although the skin was freely mobile over the mass, there was marked cutaneous dimpling (at multiple sites) on pushing the lump downward (Figure 5). Histology on the mastectomy specimen confirmed grade 2 ductal adenocarcinoma, with no tumor involvement of skin and subcutaneous fat.

Case 4

**Figure 6:**
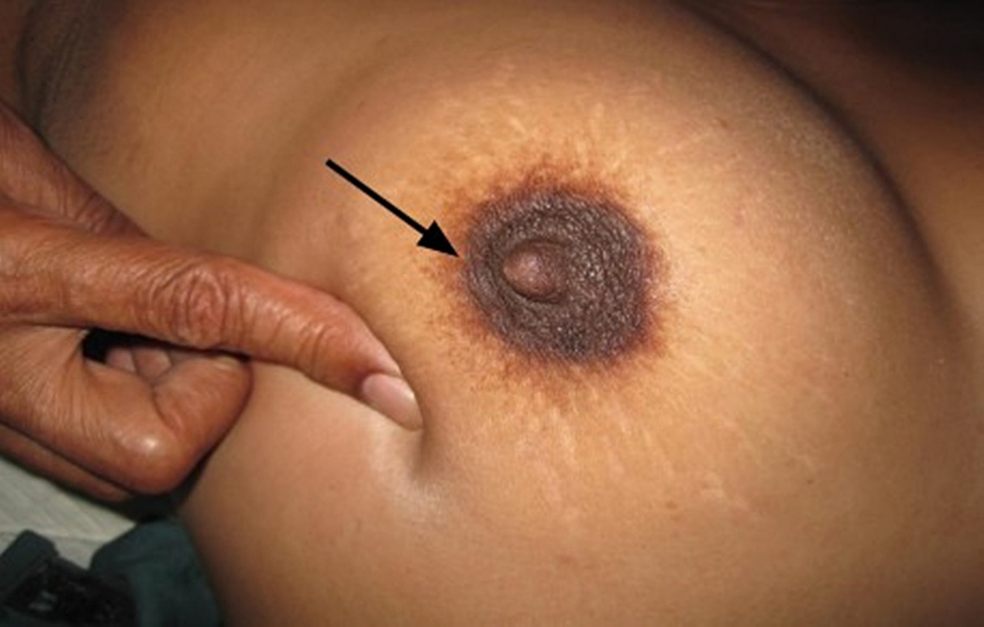
Pushing on lump shows obvious concavity (arrow)

A 38-year-old female presented with a 2 cm breast lump. There was no skin tethering on inspection or arm elevation, and the overlying skin could be pinched over it. The lump was near the lateral areola border. An obvious depression appeared over the lump on pushing it, producing a concavity at the lateral edge of the areola (Figure 6). The mastectomy specimen showed grade 2 invasive ductal carcinoma with no involvement of skin or subcutaneous fat.

Case 5

**Figure 7:**
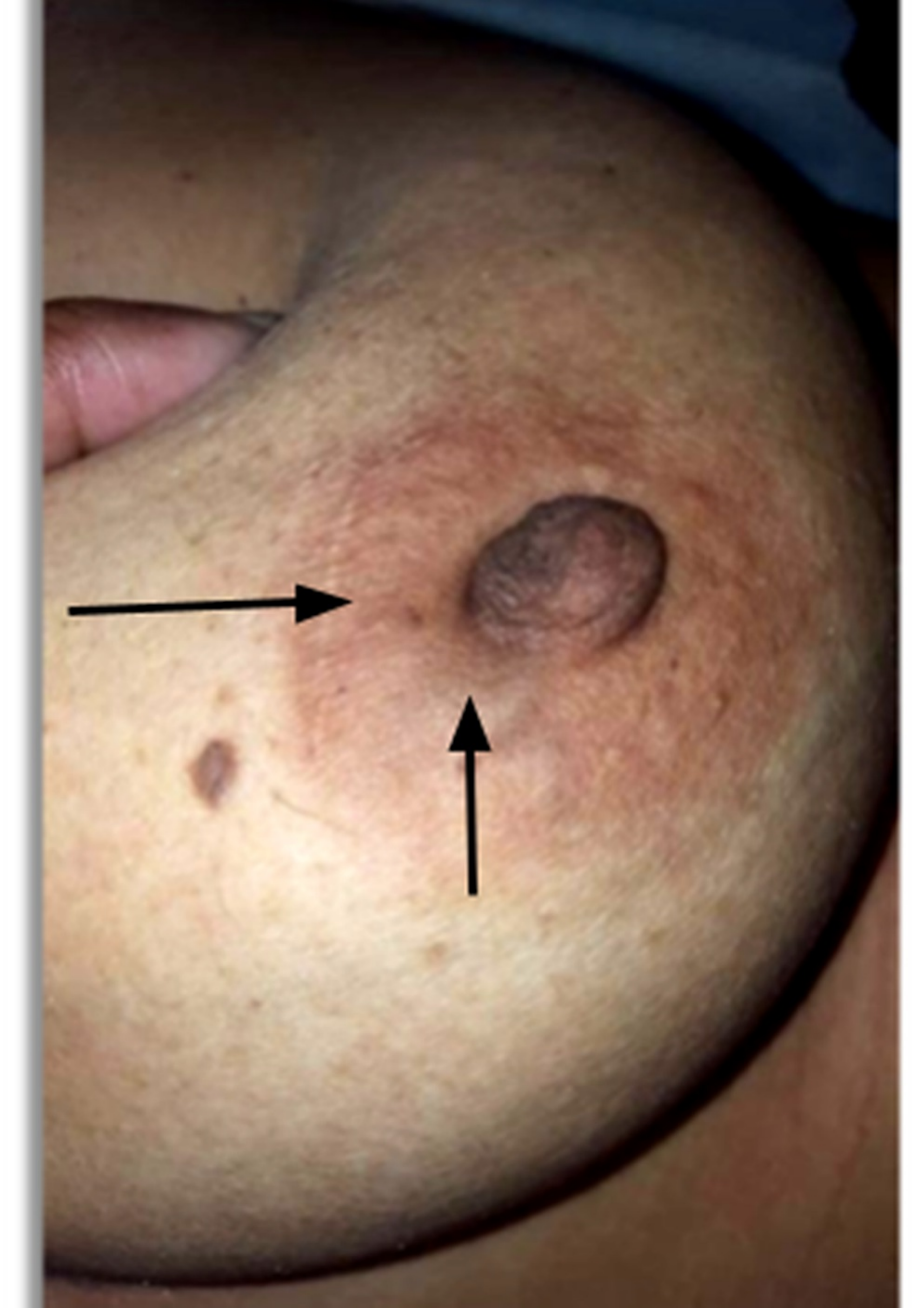
Pushing lump inferiorly produces concavity of the overlying skin (arrows)

A 40-year-old female presented with a 2 cm breast lump. She had had a fibroadenoma removed from the same breast 26 years earlier. The overlying skin was normal with no evidence of tethering on inspection or arm elevation, and the overlying skin could be pinched over it. However, on pushing the lump downward, there was an obvious concavity of the overlying skin (Figure 7). Histology on the mastectomy specimen showed grade 3 invasive ductal carcinoma with no skin invasion.

Case 6

**Figure 8:**
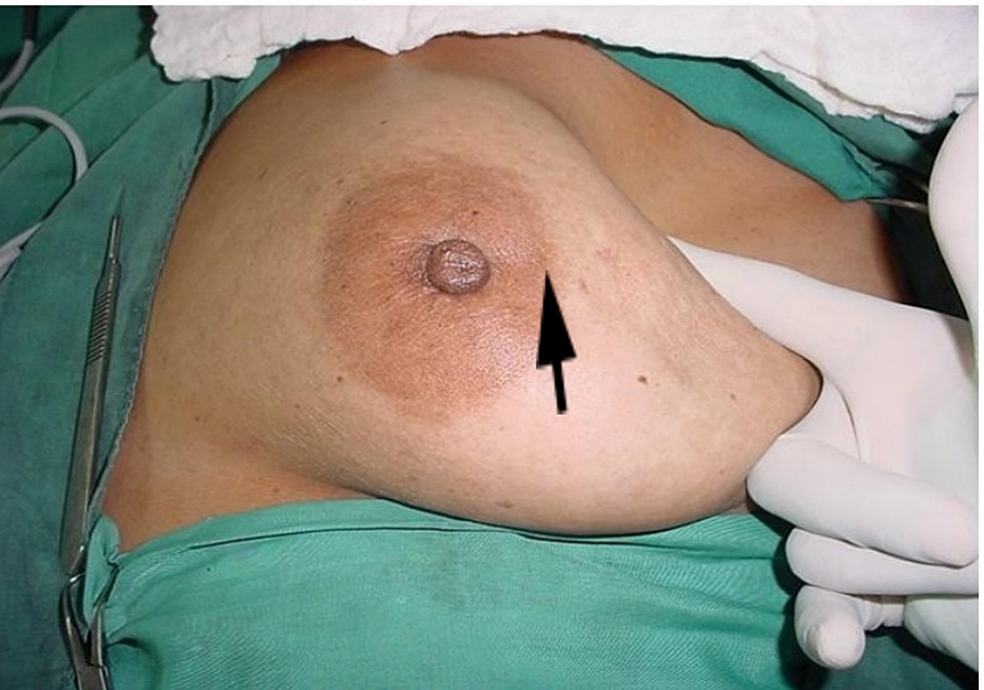
Pushing lump inferiorly shows dimpling of the skin at the lateral areolar margin (arrow)

**Figure 9:**
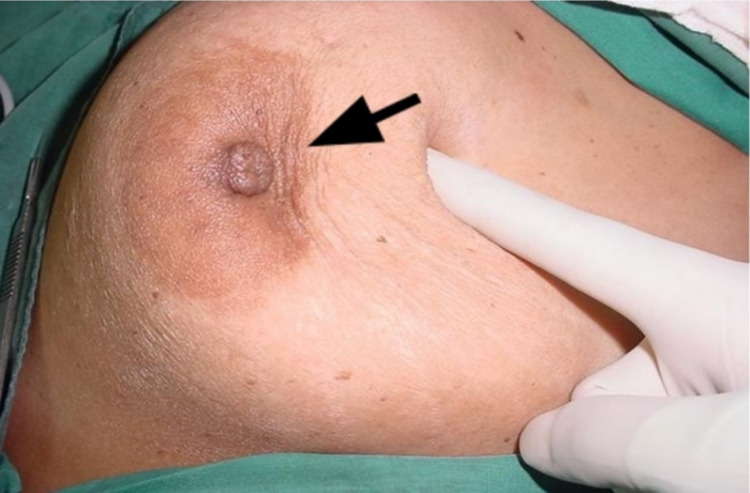
Pushing lump medially showed puckering of the areolar skin (arrow)

A 62-year-old female presented with a 3 cm left breast lump. It was freely mobile with no evidence of skin involvement on arm elevation or pinching the overlying skin. However, on pushing the lump inferiorly, a concavity developed on the skin at the lateral areola margin (Figure 8), while pushing it medially showed marked puckering of the areola skin (Figure 9). Histology on the mastectomy specimen showed grade 3 ductal carcinoma with no invasion of the skin or subcutaneous tissue.

## Discussion

Up to 45% of breast cancers are diagnosed due to a breast mass that escapes mammographic findings [7]. Clinical diagnosis is very important, especially in developing countries where radiologic investigations may not be readily available [8].

Up to 45% of breast cancers are diagnosed due to a breast mass that escapes mammographic findings [7]. Clinical diagnosis is very important, especially in developing countries where radiologic investigations may not be readily available [8]. A large study in 2011 aimed to develop and validate a clinical prediction rule where tethering was included as an independent variable on the clinical examination. All cases with skin tethering were confirmed to have breast cancer [3]. Because ST is such a "classical" sign of breast cancer, recognition in its early stages may be advantageous. In all our cases, there was no sign of ST on inspection, limb elevation, or pinching. All had normal, non-cancerous subcutaneous fat, separating the tumor from the skin (as in Figure 3), but all still had a positive pushing sign (PS).

Skin tethering on simple inspection of the breast reflects shortening of the suspensory ligaments between the skin and the tumor caused by malignant infiltration.

Cooper's ligaments, first described by Sir Astley Cooper in 1840, are fibro-collagenous septae extending from the mammary fascia of the breast stroma through the subcutaneous tissue to the inferior surface of the clavicle, the fascia over the pectoralis major, and the subdermal superficial fascia [9]. Since they attach the clavicle and pectoralis fascia to the skin through the breast tissue, these ligaments hold up and define the contour of the breast. Their stretching with aging results in drooping of the breast.

Historically, ST has been linked to the invasion of the suspensory ligaments of Cooper by the tumor. Progressive involvement of these ligaments in the malignant process draws the skin toward the tumor, resulting in skin dimpling, which may be accentuated by raising the arm upward. Lifting the arm above the head raises the clavicle and pulls on the ligaments of Cooper, which are attached to the clavicle and the pectoralis fascia. While tumor invasion may cause ST by shortening the ligaments, peritumoral fibrosis (especially in scirrhous carcinoma) may further accentuate ST without direct carcinomatous infiltration.

**Figure 10:**
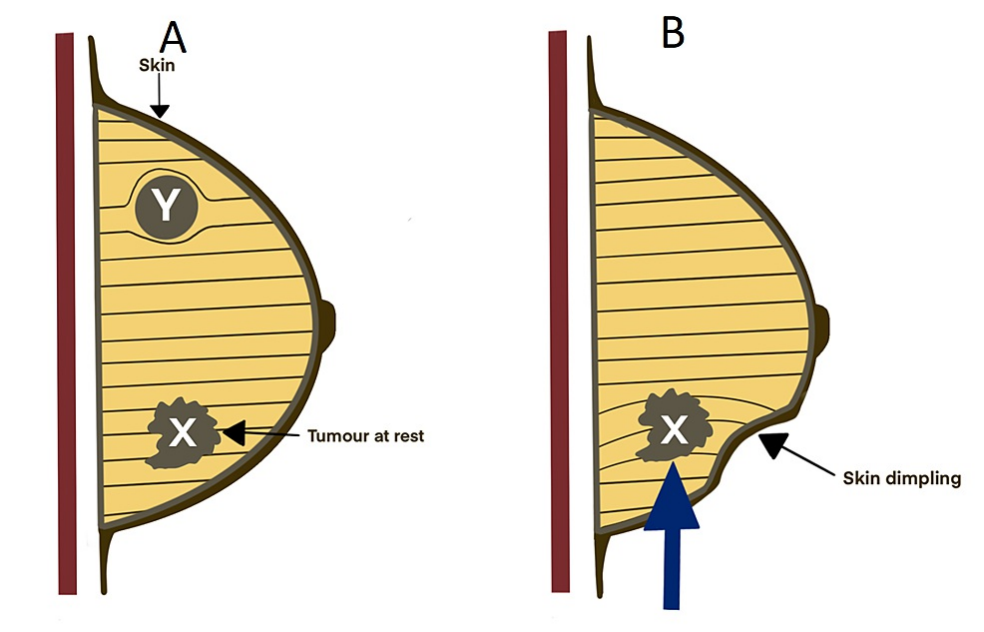
Anatomic basis of pushing sign (A) Breast carcinoma (X) at rest, attached to Cooper's ligament with no skin tethering evident. Note benign lump (Y) displaces but does not invade the ligaments. (B) Breast carcinoma pushed by the examining hand (arrow), pulls on Cooper's ligaments, causing dimpling of the overlying skin (positive pushing sign).

The pushing test (PT) displaces the tumor in relation to the surrounding parenchyma, which pulls on any involved ligaments of Cooper. In so doing, tension is transferred along with the ligaments to their attachment to the superficial fascia and the overlying skin, resulting in a positive PS (Figure 10). Any infiltration, fibrosis, rigidity, or fixity of the ligaments will result in the puckering concavity, characteristic of the PS.

In lifting the arms above the head, only an upward pull on the ligaments is exercised. During the PT, the tumor can be pushed medially, laterally, superiorly, or inferiorly, thus exerting a pull on any of Cooper’s ligaments that might be involved, allowing for the demonstration of the PS. We have noticed that the PT does not necessarily elicit a positive PS in all directions unless the disease is very advanced and other signs of ST are already present. However, a positive PS in only one direction should be enough to increase the suspicion of malignancy.

The mechanism of nipple retraction and inversion occurs similarly. Invasion of a lactiferous duct with resultant fibrosis results in tethering of the papilla mammaria, producing a slit-like appearance [10]. The involvement of multiple ducts produces an inversion of the entire papilla [10].

In cases where there is a conspicuous breast mass, ST may be easy to identify. However, the genuine advantage of the pushing sign involves inconspicuous or subtle lesions that do not present with noticeable skin changes, even with classical examination maneuvers.

The commonest lumps in the breast are benign fibroadenomas and cysts. When either of these is pushed, the overlying skin becomes convex rather than revealing the concavity of a suspected carcinoma. Fibrocystic changes usually present with pain and tenderness and involve more diffuse breast tissue changes. The clinical history also mirrors a benign path of cyclical symptoms such as the fluctuating size or heaviness of the breast. Most importantly, the disease process does not involve either the subcutaneous fat or Cooper’s ligaments; hence, tethering is usually absent [11].

The breast is comprised of a majority of adipose tissue during the non-lactating phase. When this tissue becomes non-viable due to injury or ischemia, there is scar formation. In breast fat necrosis, fibrosis may replace scar tissue [12]. These fibrotic bands extend from the necrotic tissue to the skin, presenting as tethering/nipple retraction mimicking the findings of malignancy [12]. Clinical history is particularly important as 70% of breast fat necrosis occurs after trauma, including surgery and radiation [12].

## Conclusions

Invasion of Cooper's ligaments results in a pulling force on the skin, appearing as tethering. The most common associated disease process is malignancy. The breast pushing test ensures that involvement of any of these ligaments by the tumor is thoroughly assessed apart from the other classical maneuvers. The addition of the PT to the arsenal of examination techniques may improve the detection rates of obscure lesions within the breast. A positive PS should increase the suspicion of malignancy and may indicate the need for a biopsy in the absence of suspicious radiographic findings.
